# Radiosurgery for mesial temporal lobe epilepsy following ROSE trial guidelines — A planning comparison between Gamma Knife, Eclipse, and Brainlab

**DOI:** 10.1002/acm2.12724

**Published:** 2019-09-18

**Authors:** Ganesh Narayanasamy, Steven Morrill, David Cousins, Joshua Liu, Garron Deshazer, Wesley Garner, Edvaldo Galhardo, Demitre Serletis, Jose Penagaricano

**Affiliations:** ^1^ Department of Radiation Oncology University of Arkansas for Medical Sciences Little Rock AR USA; ^2^ College Of Medicine University of Arkansas for Medical Sciences Little Rock AR USA; ^3^ Section of Neurosurgery, Children’s Hospital & Health Sciences Centre University of Manitoba Winnipeg Manitoba Canada

**Keywords:** epilepsy, epilepsy Gamma Knife, epilepsy SRS, ROSE trial, temporal lobectomy

## Abstract

**Purpose:**

This study aims to compare stereotactic radiosurgery (SRS) planning of epilepsy that complies with Radiosurgery or Open Surgery for Epilepsy (ROSE) guidelines in GammaKnife, non‐coplanar conformal (NCC) plan in Eclipse, dynamic conformal arc (DCA) plan in Brainlab, and a volumetric modulated arc therapy (VMAT) plan in Eclipse.

**Methods:**

Twenty plans targeting Mesial temporal lobe epilepsy (MTLE) was generated using GammaKnife, Eclipse with 20 NCC beams, Brainlab with 5 DCA, and Eclipse VMAT with 4 arcs observing ROSE trial guidelines. Multivariate analysis of variance and Wilcoxon signed‐rank test were used to compare dosimetric data of the plans and perform pairwise comparison, respectively.

**Results:**

The plans obeyed the recommended prescription isodose volume (PIV) within 5.5–7.5 cc and maximum doses to brainstem, optic apparatus (OA) of 10 and 8 Gy, respectively, for a prescription dose of 24 Gy. The volumes of the target were in the range 4.0–7.4 cc. Mean PIV, maximum dose to brainstem, OA were 6.5 cc, 10 Gy, 7.9 Gy in GammaKnife; 7.2 cc, 6.1 Gy, 4.5 Gy in Eclipse NCC; 7.2 cc, 6.4 Gy, 5.7 Gy in Brainlab DCA; and 5.2 cc, 8.4 Gy, 6.1 Gy in Eclipse VMAT plans, respectively. Multivariate analysis of variance showed significant differences among the 4 SRS planning techniques (*P*‐values < 0.01).

**Conclusions:**

Among the 4 SRS planning methods, VMAT with least PIV and acceptable maximum doses to brainstem and OA showed highest compliance with ROSE trial. Having the most conformal dose distribution and least dose inhomogeneity, VMAT scored higher than GK, Eclipse NCC, and Brainlab DCA plans.

## 1 INTRODUCTION

Epilepsy is the 4th most common neurological disorder in the United States with an annual incidence of more than 150,000. Mesial temporal lobe epilepsy (MTLE) refers to a chronic condition of recurrent seizure activity focally originating in the temporal lobe, namely the amygdala and hippocampus. The initial treatment for newly‐diagnosed MTLE is anti‐epileptic medication. The medical refractory MTLE cases that fail as few as two trials of medication should seek investigation for open surgery. Although seizure‐freedom rates are as high as 80–90%, very few patients are referred for resection in the United States.^1^

Stereotactic laser amygdalo‐hippocampotomy accomplishes ablation of the seizure focus with real‐time magnetic resonance thermal imaging in a minimally invasive approach that eliminates intensive care unit stay.^2^ For a subgroup of MTLE patients with medical contraindications to surgery, stereotactic radiosurgery (SRS) has emerged as an alternative therapy in the selective ablation.^3^, ^4^ Although not quite as effective compared to anterior temporal lobectomy (ATL), the preliminary results were supportive of the efficacy of SRS for select cases of MTLE. The Radiosurgery or Open Surgery for Epilepsy (ROSE) clinical trial was designed to compare the effectiveness of Gamma Knife (GK) radio surgery with lobectomy in patients with pharmaco‐resistant MTLE.^5^, ^6^ The final outcome analysis of ROSE trial suggests that both SRS and ATL have effectiveness and reasonable safety for MTLE, but ATL has an advantage in the number of seizure remission.^7^

We present here a comparison study on SRS plans in GK for treatment of epilepsy against highly non‐coplanar conformal (NCC) plan in Eclipse, dynamic conformal arc (DCA) plan in Brainlab, and a volumetric modulated arc therapy (VMAT) plan in Eclipse echoing the ROSE trial planning guidelines and dose constraints. Dosimetric comparison abiding by ROSE trial guidelines was performed using RTOG plan quality metrics.^8^ A set of primary and secondary dosimetric aims were adopted from ROSE trial guidelines in consulting among the neurosurgeon, radiation oncologists and clinical medical physicists. The primary aims of this study include: (a) prescription isodose volume (PIV) less than 7.5 cc, (b) maximal dose to brainstem of 10 Gy and maximal dose of 8 Gy to optic apparatus (OA) that includes optic nerves, optic chiasm. The secondary aims include (c) close to 100% target coverage (TC), (d) radiosurgical treatment time of less than 90 min. While every effort was made to satisfy both pairs of primary and secondary aims, the former shall be fulfilled, whereas the latter was considered less critical to this study.

Differences in dose distributions are expected from multiple sources including dose calculation algorithms, and planning techniques. There are inherent differences among these four planning modalities. Gamma Knife planning accomplishes target coverage within 50% isodose curve using multiple isocenters (shots). The maximum target dose in GK plan of twice the prescription (Rx) dose is expected to be significantly higher than those in the linac‐based plans. A SRS comparison study by Petrovic et al studied dose distributions obtained with analytical anisotropic algorithm (AAA) in Eclipse and pencil beam in Brainlab showed average dose differences of less than 3% in cranial cases.^9^ Multiple studies comparing the GK and linac‐based SRS treatments were investigated by Gevaert et al.^10^ Despite the vast differences in central dose distributions in these SRS planning techniques, clinical trials have so far failed to identify differences in treatment outcome or toxicity. In particular, it is not known if this dose differential is important in seizure remission. The lack of data on a functional target such as MTLE led us to compare the four SRS treatment deliveries.

## 2 METHODS AND MATERIALS

Stereotactic radiosurgery plans have very conformal dose distribution and steep dose gradient outside the target.^11^ Gamma Knife planning was performed in Leksell GammaPlan ver 10 using TMR 10 algorithm for treatment in a GK Perfexion unit. NCC plan was generated in Eclipse treatment planning software (TPS) (ver 11.0, Varian Medical Systems, Palo Alto, CA) utilizing AAA. Dynamic conformal arc plan based on iPlan TPS (ver 4.3.4, Brainlab, Germany) uses pencil beam algorithm for conformal dose distribution. VMAT plans in Eclipse utilizes the AcurosXB algorithm. Linac plans uses 6‐FFF MV photons for delivery in a Varian Truebeam linac with high definition 120 multi leaf collimators (HD120 MLC). Rx of 24 Gy was specified for all 4 planning techniques. The primary aims of PIV lower than 7.5 cc and maximum doses of 10 Gy to brainstem and 8 Gy to OA were followed. The secondary aims of TC close to 100% and treatment time of less than 90 min were attempted. In addition, beams with direct entrance through brainstem or OA were avoided in all linac‐based plans.

While a GK plan based on magnetic resonance images (MRI) assumes homogeneous medium, linac plans uses computed tomography (CT) for tissue heterogeneity correction. Care was taken to ensure the hot spots occur within the target volume (TV) in all the plans. All plans were created by experienced treatment planners, and reviewed by the treating radiation oncologist for plan quality and OAR dose adherence to the ROSE trial criteria.

### 2.1 Gamma Knife planning

Twenty patient cases having both MRI and CT of the whole brain were identified. This includes T1‐weighted, spoiled gradient recalled (SPGR) or magnetization prepared rapid gradient echo (MPRAGE) MRI at sub‐millimeter slice thickness. The amygdala and anterior 2 cm of hippocampus along with adjacent parahippocampal gyrus were contoured as the radiosurgical TV. Rx of 24 Gy was prescribed to the 50% isodose line using 90 degree gamma angle. GK planning was generated for treatment in a Perfexion unit allows (a) using composite shots containing combination of 4, 8, and 16 mm or blocked sectors, and (b) dynamic shaping to reduce dose to critical structures.^12^

### 2.2 Registration of CT with MRI

**Figure 1:**
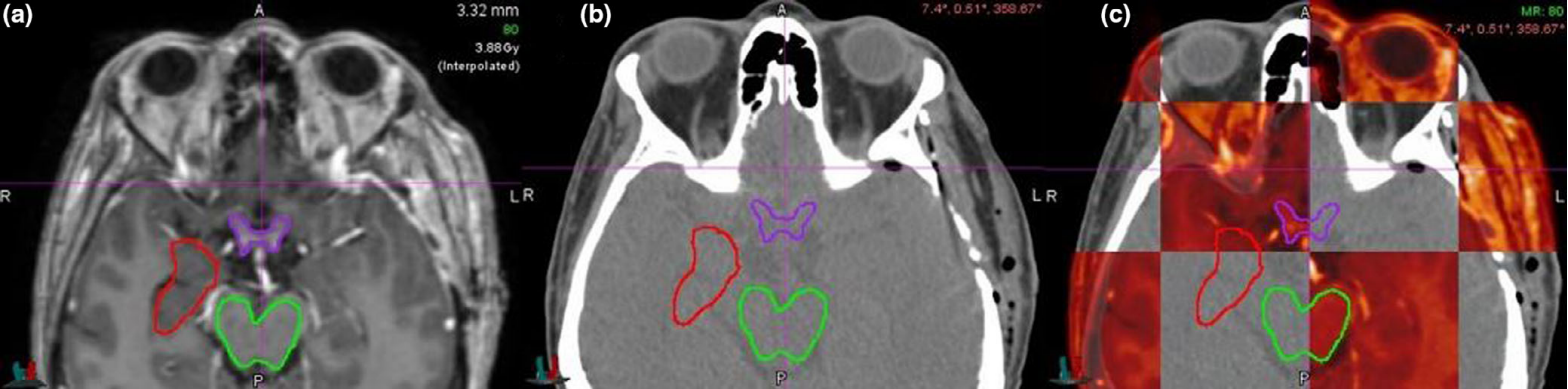
Axial slice of a representative patient showing the right epilepsy target, brainstem, and OA in (A) MRI, (B) CT, and (C) checkerboard pattern of fused MRI with CT image. MRI, magnetic resonance images; OA, optic apparatus.

The MRI along with contours, dose information and plan files were exported from Gamma Plan at 1 mm grid spacing. The MRI was rigidly registered with CT in MimVista software (ver 6.6.5, MIM Software Inc., Cleveland, OH). The contours were transferred into CT exporting to Eclipse and Brainlab TPS. Figure 1 shows the TV, brainstem, and OA in MRI and CT images.

### 2.3 Non‐coplanar conformal (NCC) plan in Eclipse

NCC plan with 20 beams employing static gantry was generated with optimum collimator angle based on the target shape along the beam’s eye view (BEV). Conformal beams were created by fitting MLCs around the target using anisotropic margin. When brainstem or OA is adjacent to the target along the BEV, a zero mm margin between the projection of MLCs and TV was used. In all other directions, a graded approach was taken to estimate the optimum margin between the projection of MLCs and TV in the range 0–2 mm for effective normal tissue sparing.^13^ On two trial cases, multiple SRS plans with margins of 0, 0.5, 1, 1.5, and 2 mm between the TV and projection of MLCs that met the ROSE trial guidelines were compared.

### 2.4 Dynamic conformal arc (DCA) plan in Brainlab

Dynamic conformal arc plan with 120^0^ gantry span and having 5 arcs (Table angles of 20°, 50°, 80°, 290°, and 315° for left target; and 50°, 80°, 290°, 315°, and 345° for right target per IEC 60601 standards) was created. Collimator angle was optimized and jaw tracking was enabled to minimize MLC leakage and in‐patient scatter. On two trial cases, an optimum Brainlab DCA plan was determined from a set of plans based on margins of 0, 0.5, 1, 1.5, and 2 mm between the TV and projection of MLCs.

### 2.5 Volumetric Modulated Arc Therapy (VMAT) plan in Eclipse

An Eclipse RapidArc plan with 4 arcs (1 being complete coplanar arc and 3 half‐ arcs with table angles of 30°, 60°, and 90° for left target; and 300°, 330°, and 90° for right target) was produced. A 1 cm wide ring was created at a gap of 5 mm from TV surface to help conform the dose. A dose normalization of 90% was used in Eclipse VMAT plans.

### 2.6 Planning Evaluation

The plan DICOM data were exported to MimVista and extracted at 10 cGy bin‐width for dosimetric comparison. We employed a few plan quality metrics recommended by International Commission on Radiation Units and Measurements (ICRU) report 83^14^ and Radiation therapy oncology group (RTOG).^8^ Conformity index (CI) is the ratio of the Rx isodose volume (*V*_RI_) to the TV.

$$\text{CI}=\frac{V_{\text{RI}}}{\text{TV}}$$

TC is that fraction of TV covered by Rx:

$$\text{TC}=\frac{\text{TV}\cap V_{\text{RI}}}{\text{TV}}$$

Dose gradient index (GI) is the ratio of 50% isodose volume to the 100% isodose volume. Although, low GI values are preferred, the acceptable range depends on TV.

$$\text{GI}=\frac{V_{50\%\text{RI}}}{V_{\text{RI}}}$$

Dose uniformity in the TV was estimated using homogeneity index (HI) based on the dose irradiated to 2% (*D*_2%_), 98% (*D*_98%_) of TV and Rx:

$$\text{HI}=\frac{\left(D_{2\%}-D_{98\%}\right)}{\text{Rx}}$$

In addition, beam‐on time and the number of shots used in GK plan and number of monitor units (MU) in linac‐based plans were tabulated in compliance with ROSE trial guidelines.

**Figure 2:**
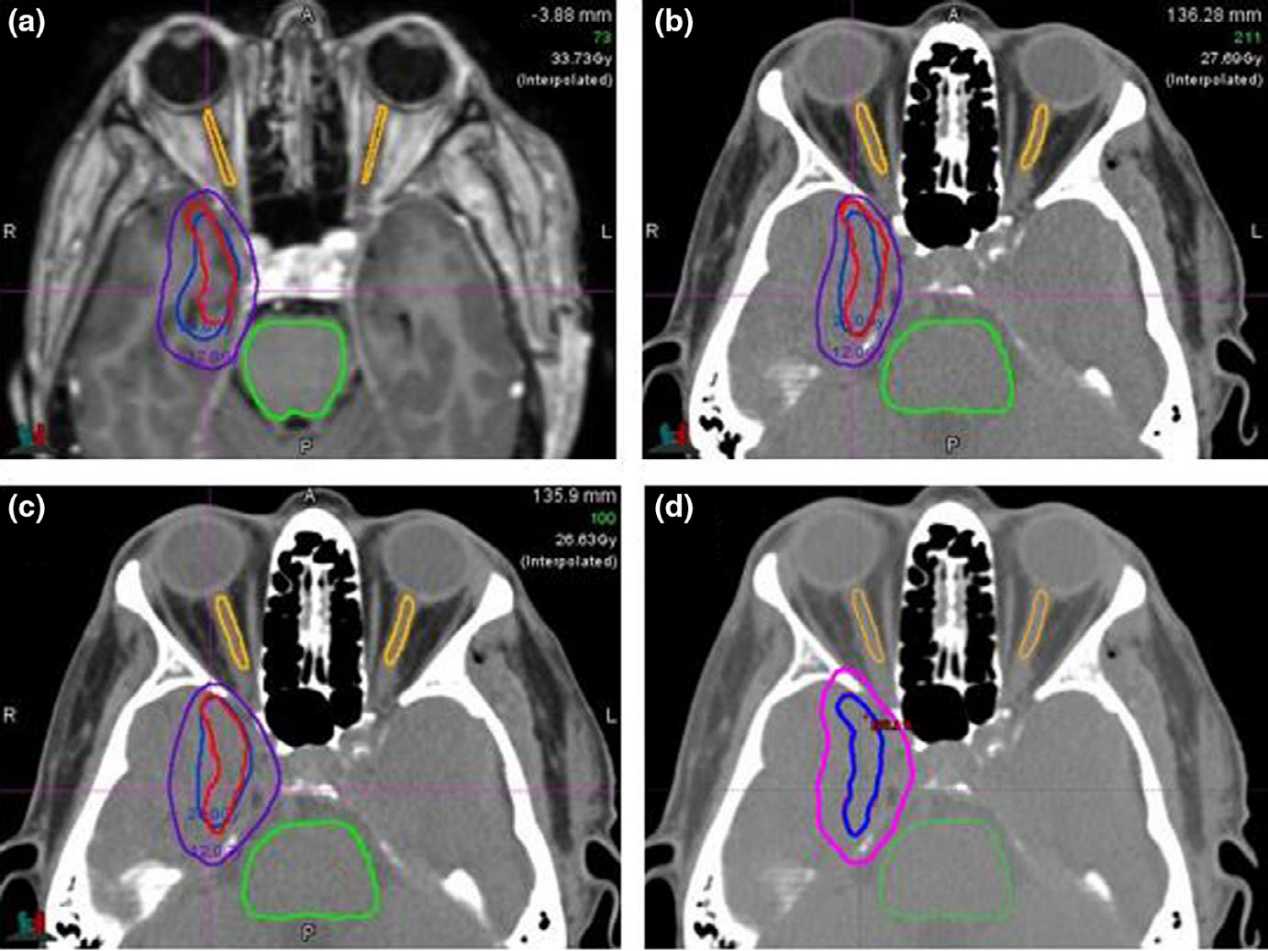
Axial slice showing the isodose distributions of 24 Gy (in blue) and 12 Gy (in purple) for a target volume (in red) (a) Gamma Knife plan, (b) Eclipse NCC plan, (c) Brainlab DCA plan, and (d) Eclipse VMAT plan. DCA, dynamic conformal arc; NCC, non‐coplanar conformal; VMAT, volumetric modulated arc therapy.

**Figure 3:**
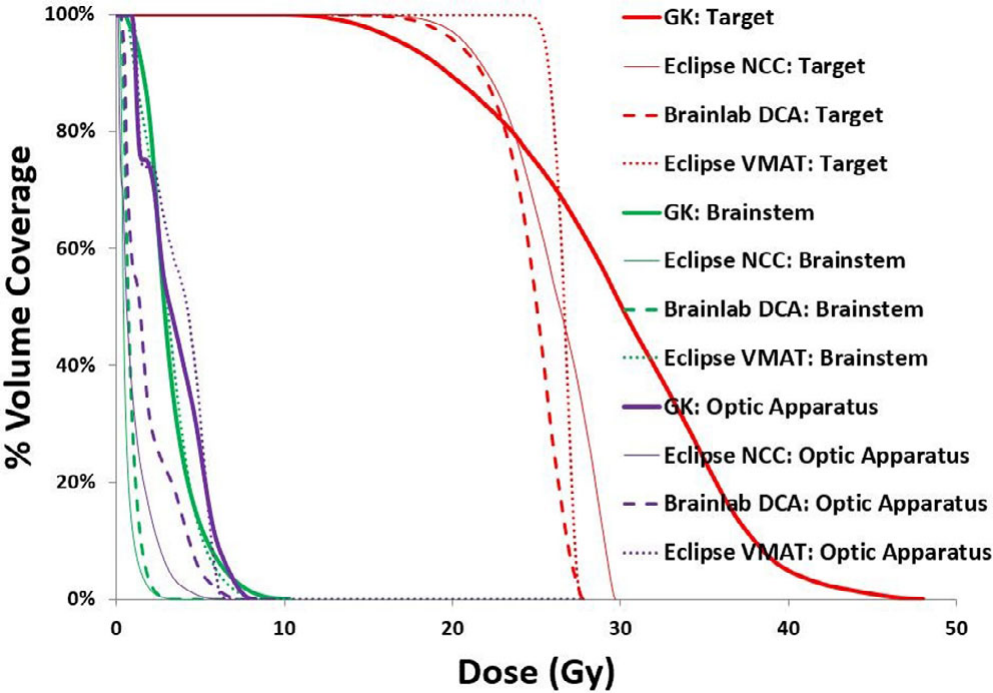
DVH of the target, brainstem and OA based on Gamma Knife (thick line), Eclipse NCC plan (thin line), Brainlab DCA plan (dashes), and Eclipse VMAT plan (dotted line). DCA, dynamic conformal arc; NCC, non‐coplanar conformal; VMAT, volumetric modulated arc therapy.

Although exit doses passing through brainstem or OA were not curtailed, none of the linac beams entered through brainstem or OA. With respect to OAR doses, 24 Gy and 12 Gy isodose volumes were displayed in Fig. 2 for a representative patient. The corresponding DVHs of the target (red), brainstem (green), and OA (purple) is shown in Fig. 3.

### 2.7 Plan statistics

Comparison of dosimetric data and plan quality metrics was based on multivariate analysis of variance (MANOVA) using STATA (ver 9.2, StataCorp, College Station, TX). Data normality was tested using Shapiro–Wilk test. Pairwise comparison analysis uses two‐tailed paired Student’s T‐test or Wilcoxon signed‐rank test. P‐value less than 0.0125 was considered statistically significant in pairwise comparisons.

## 3 RESULTS

The target volumes were in the range 4.0–7.4 cc with a mean ± *SD* of 5.5 ± 1.0 cc. Overall beam‐on time was 86 ± 17 mins in GK plans using 11.5 ± 4 (range: 5–20) shots which fits well within ROSE trial guideline of 6–30 shots.

### 3.1 Treatment margins

On two trial patient plans, Eclipse NCC, Brainlab DCA, and Eclipse VMAT plans were created using 0, 0.5, 1.0, 1.5, and 2 mm margin between the projection of MLCs and TV. It was observed that 0 mm plan could spare the OARs and stay within the prescribed range for PIV but has poor TC. On the other hand, the plan with 2 mm margins provided higher TC but breached the upper limit of PIV and maximum OAR doses. An optimum 1 mm margin between the projection of MLCs and TV was used on all linac‐based plans.

### 3.2 Planning comparison

**Figure 4:**
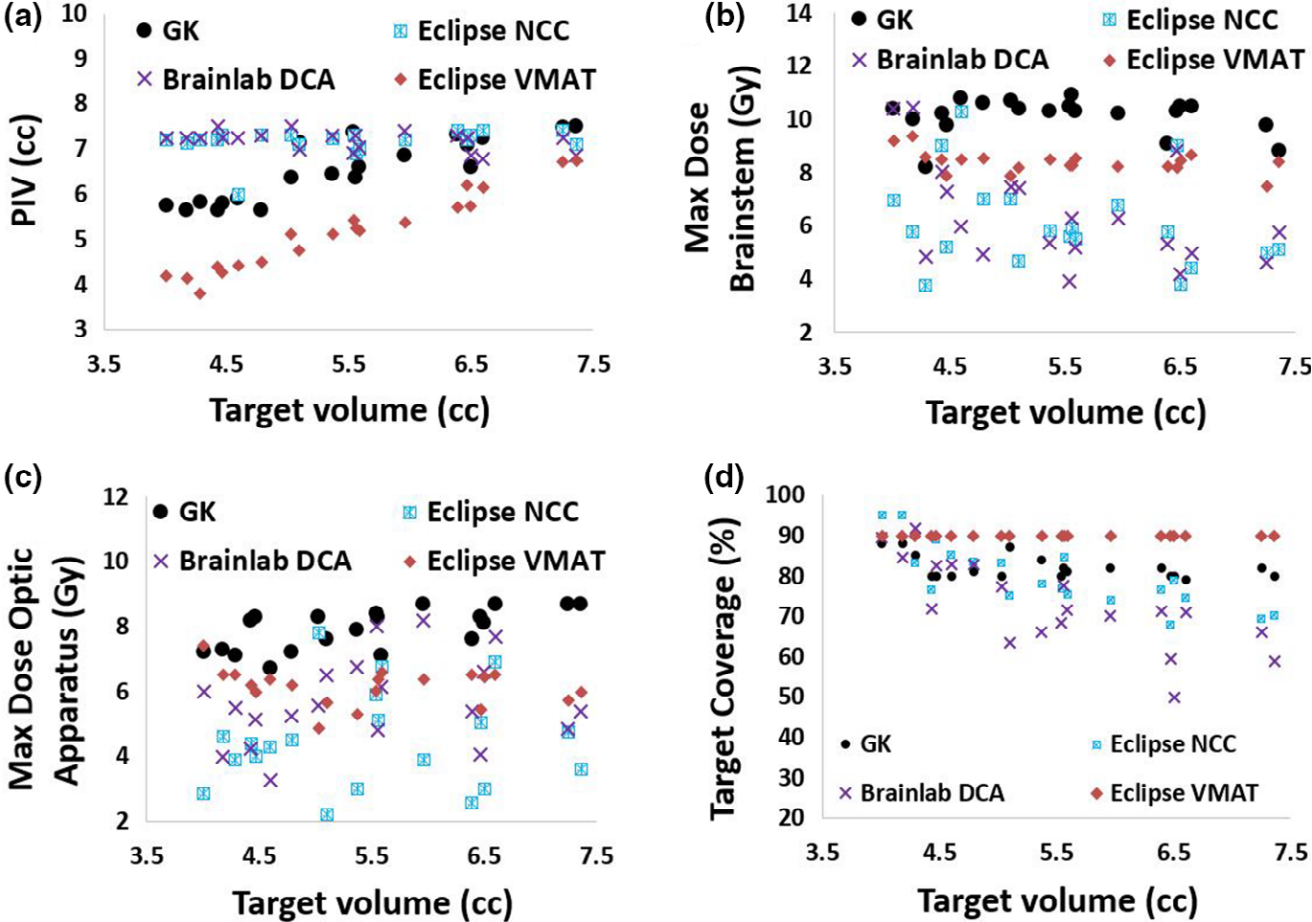
Results of primary aims of this study — PIV, maximum doses to brainstem and optic apparatus, and TC. Notice Eclipse VMAT plans have statistically significant lowest PIV and highest TC, the maximum doses to OARs are acceptable. PIV, prescription isodose volume; TC, target coverage; VMAT, volumetric modulated arc therapy.

The mean ± *SD* of target dose, OAR doses and plan quality metrics tabulated in Table 1 show significant differences among the four SRS planning techniques that observed ROSE trial dosimetric criteria. Prescription isodose volume was significantly low in Eclipse VMAT plans than other three plans, GK plans came second lowest, whereas Eclipse NCC and Brainlab DCA plans were not different. With regard to the maximum point dose to brainstem, Eclipse NCC, and Brainlab DCA were the lowest followed by Eclipse VMAT, and GK plans had the highest values. The maximum point dose to the OA was found to be significantly increasing in going from Eclipse NCC plans to Brainlab DCA, Eclipse VMAT, and GK plan. However, the difference in maximum OA dose between Brainlab DCA and Eclipse VMAT plans are marginally significant with *P*‐value = 0.015. TC was highest in Eclipse VMAT plans followed by GK plans and Eclipse NCC plans, and lowest in Brainlab DCA plans. The maximum target dose was significantly high in GK plan as expected and decreases in the following order: Eclipse NCC, Brainlab DCA, and Eclipse VMAT plans. Volume of the 50% isodose line (V12Gy) was estimated to be significantly increasing in going from GK to Eclipse VMAT to Eclipse NCC to Brainlab DCA plans. Figure 4 exhibits the PIV (cc), maximum brainstem dose (Gy), maximum OA dose (Gy), and TC (%) as a function of target volume (cc).

**Table 1:** Statistical summary of dosimetric comparison among the four SRS planning techniques expressed as mean ± *SD*

	GK	Eclipse NCC	Brainlab DCA	Eclipse VMAT
PIV (cc)	6.5 ± 0.7	7.2 ± 0.3*	7.2 ± 0.2*	5.2 ± 0.9*^,+,$^
Max brainstem dose (Gy)	10 ± 0.7	6.1 ± 1.7*	6.4 ± 1.9*	8.4 ± 0.4*^,+,$^
Max optic apparatus dose (Gy)	7.9 ± 0.6	4.5 ± 1.5*	5.7 ± 1.3*^,+^	6.1 ± 0.6*^,+^
Target coverage (%)	82.1 ± 2.8	79.6 ± 7.7	72.8 ± 10.8*^,+^	90 ± 0*^,+,$^
Max target dose (Gy)	48 ± 0	30 ± 1.3*	28 ± 1.1*^,+^	25.7 ± 0.7*^,+,$^
Volume of 12 Gy(cc)	21.9 ± 3.1	26.5 ± 2.7*	31.5 ± 3.7*^,+^	25.3 ± 3.6*^,+,$^
CI	1.2 ± 0.1	1.3 ± 0.2*	1.3 ± 0.3*	0.9 ± 0.0*^,+,$^
GI	3.4 ± 0.3	3.7 ± 0.4*	4.4 ± 0.6*^,+^	4.9 ± 0.4*^,+,$^
HI	1.1 ± 0.1	0.4 ± 0.1*	0.3 ± 0.1*^,+^	0.1 ± 0.0*^,+,$^
MU	NA	4760 ± 463	4688 ± 366	7741 ± 1057^+,$^

DCA, dynamic conformal arc; NCC, non‐coplanar conformal; PIV, prescription isodose volume; VMAT, volumetric modulated arc therapy.

Note that significant differences against GK, Eclipse NCC, Brainlab DCA plans are specified as *, ^+^, and ^$^ respectively.

**Figure 5:**
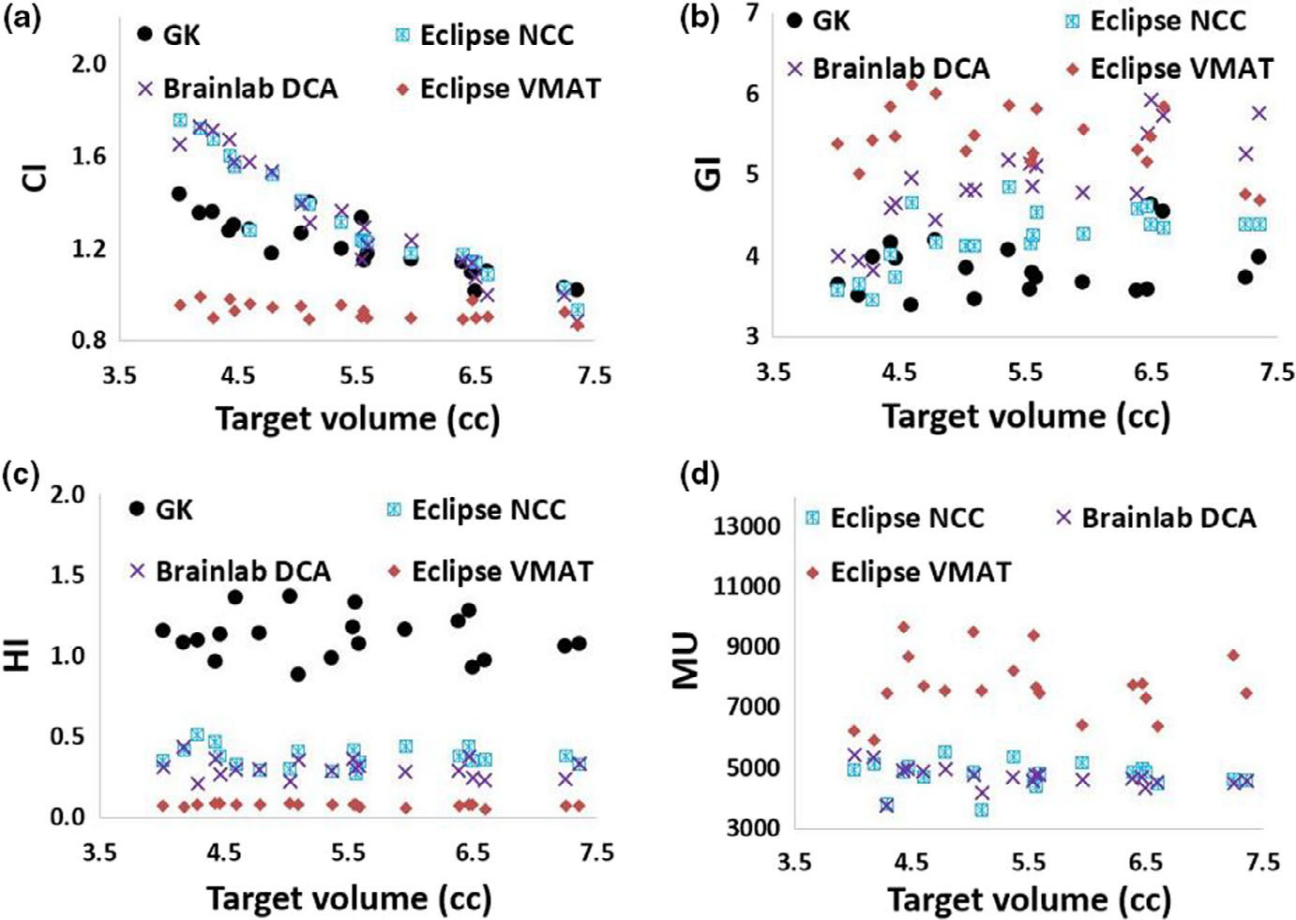
CI, GI, HI, and MUs of the 20 plans across the four SRS planning techniques. The Eclipse VMAT plans had significantly lower CI, higher GI, and lower HI than the other three SRS plans. SRS, Stereotactic radiosurgery; VMAT, volumetric modulated arc therapy.

Displayed in Fig. 5 are the plan quality metrics. The respective mean ± *SD* of CI, GI, HI values are 1.2 ± 0.1, 3.4 ± 0.3, 1.1 ± 0.1 in GK; 1.3 ± 0.2, 3.7 ± 0.4, 0.4 ± 0.1 in Eclipse NCC; 1.3 ± 0.3, 4.4 ± 0.6, 0.3 ± 0.1 in Brainlab DCA; 0.9 ± 0.0, 4.9 ± 0.4, 0.1 ± 0.0 in Eclipse VMAT plans. From [Fig. 5(a)], it is interesting to note that CI has a decreasing trend with target volume with Pearson correlation coefficient, R^2^ of 0.93 in Brainlab DCA, 0.88 in Eclipse NCC, and 0.77 in GK plan. Eclipse VMAT plans provide better dose conformity than GK, Eclipse NCC and Brainlab DCA plans, whereas the latter two were not significantly different. Computed values of GI was significantly increasing from GK to Eclipse NCC to Brainlab DCA to Eclipse VMAT plans. The Brainlab DCA plans have an increasing GI values with target value with R^2^ of 0.68 in [Figure 5(b)]. On the other hand, values of HI were found to be significantly decreasing from GK to Eclipse NCC to Brainlab DCA to Eclipse VMAT plans. This can be explained by the sharp dose differential in GK plans and the most homogeneous dose distribution in Eclipse VMAT plans. Number of MUs in Eclipse NCC (4760 ± 463) and Brainlab DCA plans (4688 ± 366) did not display significant differences, and both were significantly lower than MUs of Eclipse VMAT plan (7741 ± 1057).

## 4 DISCUSSION

Limited temporal lobe resection for MTLE has been shown to result good treatment outcomes.^15^, ^16^ A review article by McGonigal et al. states SRS was an efficacious treatment to control MTLE seizures and possesses a better risk‐benefit ratio than surgical methods.^17^ In the prospective trial by Barbaro et al.^3^ involving 30 MTLE patients, Rx of 24 Gy showed improved seizure control with earlier remission than 20 Gy. A uniform Rx of 24 Gy was used in this study.

In a 24 patient study, the authors concluded that SRS‐related neuropsychological morbidities were not substantially different from those of open surgical resection of the temporal lobe.^18^ From the preliminary results of ROSE trial presented at the 2016 American Epilepsy Society Annual Meeting, the study was underpowered to show the noninferiority of GK.^19^, ^20^ More patients were seizure‐free during the last year of the trial (78% in surgery versus 52% in GK arm). Most patients in both groups had no or minimal changes in verbal memory. Quality of life measures improved rapidly for those who received open surgery, and slowly for those in the GK arm. Incidence and severity of visual field defects after GK are similar to resection.^21^

This study was conducted to quantitatively evaluate PIV, critical organ dose, target coverage, dose conformity, dose heterogeneity, and dose gradient among four SRS planning techniques used in our institution. Due to a large range of planning parameters and methodologies used, we conducted comparison between the GK plan, Eclipse non‐coplanar conformal plan, Brainlab dynamic conformal plan, and Eclipse VMAT plan.

One of the main aims of this study is to irradiate lowest volume of the 24 Gy isodose or PIV that should typically be less than 7.5 cc, a ROSE trial recommendation following observations made in the multicenter study by Barbaro et al.^3^ In certain scenarios, meeting the secondary aims of close to 100% TC would necessarily violate the volumetric constraint on PIV. In case, high TC were in contradiction with the primary aims, the latter was preferred. The dose normalization of 50% in GK and 90% in Eclipse VMAT, but variable in Eclipse NCC and Brainlab DCA plans could be attributed to variation in TC. It is pertinent to state here that any further improvement in TC in Eclipse NCC or Brainlab DCA plans would have breached the ROSE trial recommended upper limit of the PIV.

In our study, CI and PIV were significantly lower and TC is higher in Eclipse VMAT plans than any of the other SRS plans, as displayed in Table 1. According to the Stanford experience with SRS of resected cavities, higher conformity index correlates with lower rate of tumor recurrence.^22^ However, the necessity of having a high conformity and TC is not fully established in a functional target like MTLE.

Balagamwala et al found a strong correlation between HI and GI values which implies more homogeneous plans (low HI) tend to have more gradual dose falloffs outside the target (high GI).^23^ The authors observed that patients did not develop toxicity when CI ≤ 2, maximum target dose ≤ twice Rx, and GI ≥ 3. Gevaert et al had concluded that GK Perfexion‐based SRS plan can achieve high conformity while minimizing the low‐dose spread.^24^ GI values were lower in GK yielding significantly more heterogeneous dose distribution in GK than linac‐based plans.^25^ Although steep dose fall‐off is preferred in SRS, there is no clinical evidence supporting the belief that target dose homogeneity is detrimental to disease control,^26^, ^27^ even more so in functional targets such as MTLE. With least HI and highest GI values, Eclipse VMAT plans stands apart from the other three SRS planning techniques studied here.

Low‐dose spillage to healthy brain parenchyma is a concern for radiation‐induced malignancy and necrosis. The V12Gy (associated with neurotoxicity^28^) being significantly low in GK plans, increases in Eclipse VMAT, Eclipse NCC, and a large spread of low isodose volume was observed in Brainlab DCA plans.^29^ The maximum point doses to brainstem and OA are lowest in the Eclipse NCC plans and highest in GK plans. Maximum dose limits to brainstem and OA stipulated in ROSE trial are considered conservative, and the incidence of optic neuropathy is rare for a maximum dose of < 8 Gy and presents low risk for maximum dose 10–12 Gy.^30^ With regard to brainstem, maximum dose of 12.5 Gy is associated with a < 5% risk of cranial neuropathy in SRS.^31^ Thus, we believe that the dosimetry of MTLE plans can be improved using evidence‐based tolerance doses to brainstem and OA which can be higher, while respecting PIV constraint.

SRS plans for MTLE that met the ROSE trial guidelines were created using the four planning techniques and a few features stood out. A PubMed search with keyword combinations that include “functional,” “tumor,” “stereotactic,” and “SRS plan” failed to return any tangible result making this possibly the first comparative study on SRS of a functional target. Our study has the following limitations: (a) did not address the correlation of a conformal dose distribution with treatment outcome in MTLE, (b) was not designed to analyze the importance of low‐dose spread or dose gradient with seizure‐free survival, (c) did not include hypo‐fractionation, and (d) did not address the correlation of target inhomogeneity with treatment outcome in MTLE.

## 5 CONCLUSIONS

All four SRS planning techniques (GK, Eclipse highly non‐coplanar beams, Brainlab dynamic conformal arcs, and Eclipse VMAT plan) met the ROSE trial criteria for MTLE. MANOVA test confirms that the four SRS planning techniques yield significantly different dosimetric and plan quality metrics (*P*‐value < 0.0125). Eclipse VMAT plans came out superior with least prescription isodose volume, higher target coverage, least of both dose conformity and dose homogeneity indices, while respecting maximum point doses to brainstem and optic apparatus. Considering the outcome of dose differential remains unknown for a functional target (such as epilepsy), Eclipse VMAT shall be considered as a viable option for SRS of MTLE.

## CONFLICT OF INTEREST

The authors declare no conflict of interest.
